# Influence of age, sex, bodyweight, and training on echocardiographic measurements in healthy mixed-breed donkeys

**DOI:** 10.1007/s11259-024-10461-6

**Published:** 2024-07-05

**Authors:** Chiara Bozzola, Ilaria Guffanti, Asia Ortolina, Valerio Bronzo, Enrica Zucca

**Affiliations:** 1 Department of Veterinary Medicine and Animal Science, Università degli Studi di Milano, Lodi, Italy; 2 Como, Italy; 3 Pavia, Italy

**Keywords:** Donkey, Echocardiography, Cardiac measurements, M-Mode, B-Mode

## Abstract

The donkey has always been a notable working animal and its importance as a companion animal has been growing over the last few years. However, there are only a few studies about cardiology in this species. Therefore, this study aimed to evaluate the influence of age, sex, training, and bodyweight on cardiac dimension in mixed-breed donkeys. Forty-five clinically and para-clinically healthy mixed-breed donkeys were included, and B-mode and M-mode echocardiographic measurements were recorded. Multivariate linear regression analysis was performed to identify the effect of age, sex, bodyweight, and training on cardiac measurements. Most of the echocardiographic measurements showed a strong statistically significant correlation with bodyweight. Linear regression analysis between echocardiographic measurements and bodyweight was performed to obtain the values of the intercept and slope of the linear equation to calculate the echocardiographic measurements as a function of bodyweight. This is the first study reporting a strong linear correlation between echocardiographic measurements and bodyweight and reporting a correlation between training and echocardiographic parameters in donkeys, suggesting that this variable should be considered when assessing cardiac dimensions in these animals.

## Introduction

The donkey (*Equus asinus*) is a widespread animal, derived from the African wild donkey, that has lived alongside humans since the domestication process started, approximately 10,000 years ago. In developing countries, it represents an essential resource for agriculture and the transport of people and goods. In developed countries, it is mainly kept as a companion animal or used for onotherapy, bred for milk production, and sometimes employed in athletic activities (Raw et al. 2021).

Despite his importance as a working or companion animal, there are only a few studies in the literature about cardiovascular diseases (Roberts and Dukes-McEwan 2016a) and echocardiographic examination (Amory et al. 2004; Hassan and Torad 2015; Roberts and Dukes-McEwan 2016b; Farag and Ibrahim 2020; Cruz-Aleixo et al. 2023). Some of these studies were conducted on purebred donkeys, but in Italy, in addition to small populations belonging to a specific breed (such as Amiata, Asinara, Martina Franca, Ragusano, Romagnolo, Pantesco, Sardo, and Viterbese), the majority of donkeys are mixed-breeds with extreme variability in bodyweight (BW) and size. Due to the stoic nature of donkeys and their lower athletic demand (compared to horses), in association with a lower prevalence of audible heart murmurs (2%) (Roberts and Dukes-McEwan 2016a), the diagnosis of cardiac diseases relies on reference values for echocardiographic measurements (Hassan and Torad 2015).

In horses, the influence of breed (Bakos et al. 2002), age (foals or adults) (Lombard et al. 1984), bodyweight (Al-Haidar et al. 2013, 2017), and training (Buhl et al. 2005) on echocardiographic measurements has been reported. However, studies regarding the influence of these parameters on donkeys are limited (Amory et al. 2004; Hassan and Torad 2015; Roberts and Dukes-McEwan 2016b; Farag and Ibrahim 2020).

Therefore, the aim of this study, which was conducted on a population of healthy mixed-breed donkeys, was to evaluate the influence of age, sex, bodyweight, and training on cardiac dimensions.

## Materials and methods

### Donkeys

Client-owned donkeys of different age, sex, breed, and bodyweight were voluntarily enrolled in this study. All the donkeys lived on different farms in northern Italy (45°47’91” N, 9°84’52” E). The inclusion criteria were the absence of abnormalities based on history, hematology, clinical examination, electrocardiography, and echocardiography. The exclusion criteria were the presence of any significant echocardiographic morphological abnormalities or valvular regurgitation detected by Color Flow Doppler Echocardiography (CFD). Donkeys that were noncompliant with handling were also excluded. All animals were evaluated at their stable or in the paddock. The Body Condition Score (BCS) and bodyweight were calculated for all donkeys. The BCS was estimated using a five-degree classification (Svendsen 2008a). BW was estimated using a diagram that associates the heart girth and the height measurements (Svendsen 2008b). Information about deworming, vaccination, nutrition, and use were also recorded.

### Echocardiography

**Fig. 1:**
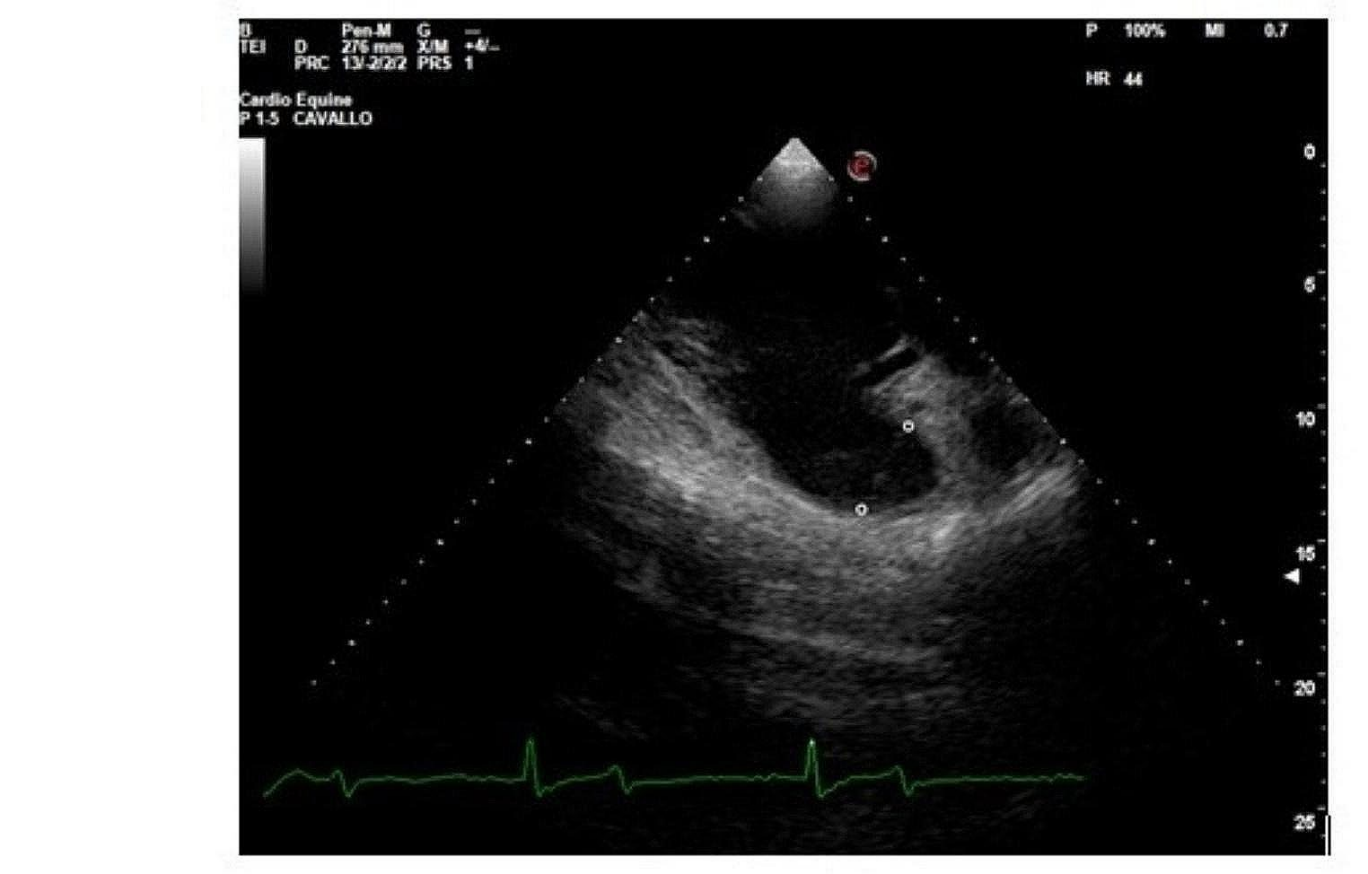
Right parasternal right ventricular inflow-outflow view showing the measurement of the pulmonary artery valve diameter - PAD (°) at end-diastole

**Fig. 2:**
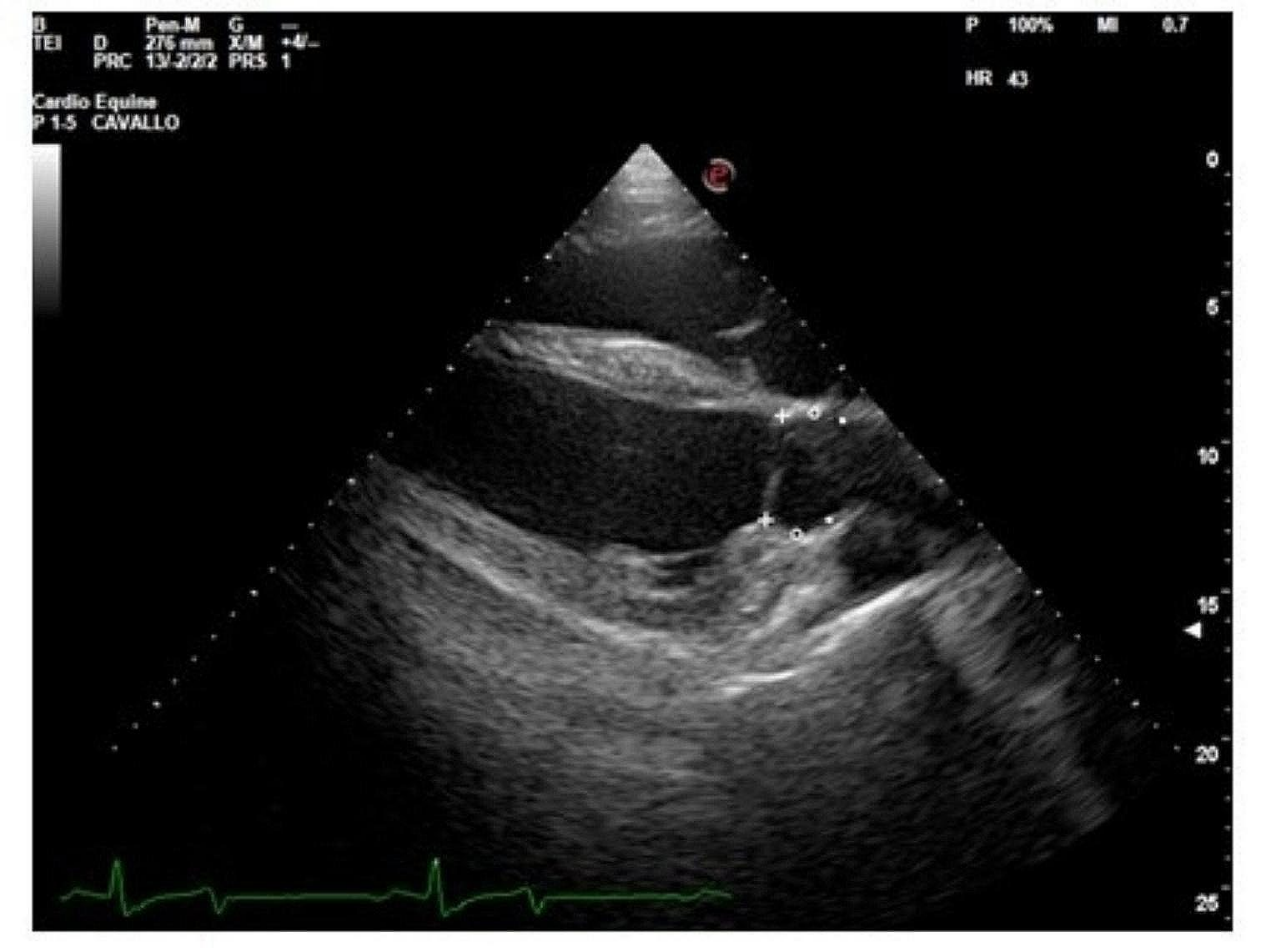
Right parasternal left ventricular outflow tract view showing the measurements of the aortic diameter at the base of the valve - ABS (+), the sinus of Valsalva level – ASV (°), and the sino-tubular junction - AJT (▪) at end-diastole

Echocardiography was performed in standing and non-sedated donkeys, held by their owners using only a halter, using an Esaote MyLab Omega VET ultrasound machine with a 2.5 MHz phased array transducer. To increase contact between the probe and the skin, alcohol was used. Cineloops of the right parasternal long axis and short axis views and left parasternal long axis views (Long et al. 1992), were recorded for offline analyses. An electrocardiogram was recorded simultaneously using a base-apex lead. Cardiac measurements were obtained in two-dimensional (B-mode) and mono-dimensional (M-mode) images. End-diastolic measurements were acquired at the onset of the QRS complex. End-systolic measurements were acquired at the level of the maximum excursion of the interventricular septum. CFD was used to detect any valvular regurgitation. The pulmonary artery diameter (PAD) at end-diastole was measured from the right parasternal right ventricular inflow-outflow view at the level of the pulmonary artery valve (Fig. 1). End-diastolic aortic diameters were measured from the right parasternal left ventricular outflow tract view at the base of the valve (ABS), the sinus of Valsalva level (ASV), and the sino-tubular junction (AJT) (Patteson et al. 1995) (Fig. 2). The aortic root diameter (AOD), pre-ejection period (PEP), and ejection time (ET) were measured by M-mode echocardiography of the aorta acquired from the right parasternal short axis view. The septal-E point separation (EPSS), which was measured from the peak of the E wave to the maximal excursion of the interventricular septum, was obtained on M-mode echocardiography, from the right parasternal short axis view of the left ventricle at the mitral valve level. The right ventricular internal diameter in diastole (RVIDd) and systole (RVIDs), interventricular septal thickness in diastole (IVSd) and systole (IVSs), left ventricular internal diameter in diastole (LVIDd) and systole (LVIDs), and left ventricular free-wall thickness in diastole (LVFWd) and systole (LVFWs) were acquired from the M mode of the right parasternal short axis view of the left ventricle at the chordal level. Fractional shortening (FS) was calculated according to the following formula: FS = [(LVIDd – LVIDs)/LVIDd]x100. The end-diastolic left atrial diameter (LAD) and the mitral valve diameter (MVD) were measured from the left parasternal long-axis view. At least three cardiac cycles were measured and the mean value for each parameter was obtained.

### Statistical analyses

All the statistical analyses were performed using IBM SPSS 29.0 (IBM, SPSS, Armonk, NY, USA). The data distribution was evaluated using the Shapiro‒Wilk test. Since the data were not normally distributed, the quantitative variables were reported as median (minimum-maximum). Qualitative variables were reported as relative frequencies. A descriptive analysis for echocardiographic measurements, including the mean, standard deviation (SD), 95% Confidence Interval (CI) for the mean, minimum and maximum values, was performed. Multivariate linear regression analysis was used to identify the effect of age, sex, bodyweight, and training on the echocardiographic measurements in donkeys. The level of significance was defined as *p* < 0.05. Linear regression analysis between echocardiographic measurements and bodyweight was performed to obtain the values of the intercept (a) and slope (b) of the regression line. These values were used to calculate the echocardiographic measurements (Y) as a function of body weight (X), using the following linear equation: $Y=bX+a$.

## Results

### Donkeys

Sixty mixed-breed donkeys of different age, sex, and body weight were evaluated. According to the inclusion and exclusion criteria, 7 were excluded due to a lack of collaboration during handling, 5 donkeys were excluded due to the presence of valvular abnormalities or/and regurgitation, and 3 jennies were excluded because pregnant.

Therefore, 45 animals were included in this study, 25 were females (56%), 12 were males (27%) and 8 were geldings (17%). The median age was 5 years (8 months – 24 years); and the median body weight was 200 kg (76–395 kg). The median heart rate (HR) was 46 bpm (reference values: 36–68 bpm), and the median packed cell volume (PVC) was 35.3% (reference values: 27–42%) (French and Patrick 1995; Burden et al. 2016). All donkeys had a BCS of 3/5. All animals were regularly dewormed and vaccinated. Regarding activity, 12 donkeys (27%) underwent regular training for race competitions in the context of traditional town events (one hour of training, including walking, trotting, and galloping, two/three days per week). All the animals were fed with hay and had access to the paddock for a few hours per day. Mash was also given to the donkeys in training.

### Statistical analyses

Descriptive statistics for echocardiographic measurements, including the mean, SD, 95% CI for the mean, minimum and maximum values, are reported in Table 1. The results of multivariate linear regression are reported in Table 2. Except for PEP, ET, EPSS, RVIDd, RVIDs, LVFWs, and FS%, the echocardiographic measurements showed a statistically significant correlation with bodyweight. For LVIDs and IVSd, a statistically significant correlation with sex was found. The training was significantly correlated with the AJT, AOD, RVIDd, LVFWs, and MVD. None of the echocardiographic measurements were significantly correlated with age. The results of the linear regression between echocardiographic measurements and bodyweight, including the coefficient of determination, *p*-value, mean value, and 95% CI of the intercept and slope are reported in Table 3.

**Table 1:** Results of descriptive statistics of echocardiographic measurements of cardiac dimensions in mixed-breed donkeys

Measurement	Mean	SD	95% Confidence Interval for the means	Minimum	Maximum
*B-mode*					
PAD (mm)	34.463	6.010	31.925–37.000	23.5	45.3
ABS (mm)	37.779	5.657	35.390–40.168	28.6	46.5
ASV (mm)	44.475	6.363	41.788–47.162	34.7	56.0
AJT (mm)	36.683	6.091	34.112–39.255	26.9	47.1
MVD (mm)	64.879	8.054	61.478–68.280	51.4	79.7
LAD (mm)	72.950	9.275	69.033–76.876	55.3	87.1
*M-mode*					
AOD (mm)	43.167	6.442	40.446–45.887	29.8	53.1
PEP (ms)	102.750	27.155	91.280–114.220	60.0	168.0
ET (ms)	424.250	63.034	397.63–450.87	258.0	504.0
EPSS (mm)	2.169	1.476	1.545–2.792	0.0	5.1
RVIDd (mm)	24.496	4.962	22.401–26.591	14.2	34.2
RVIDs (mm)	15.967	5.451	13.665–18.268	4.5	30.5
IVSd (mm)	19.875	3.689	18.317–21.433	13.8	26.9
IVSs (mm)	32.900	4.955	30.808–34.992	23.0	40.2
LVIDd (mm)	70.354	13.330	64.725–75.983	43.6	88.7
LVIDs (mm)	37.154	9.077	33.321–40.987	21.9	54.5
LVFWd (mm)	16.462	3.152	15.132–17.793	12.6	24.7
LVFWs (mm)	23.842	4.910	21.769–25.915	16.6	33.4
FS (%)	47.511	5.738	45.088–49.934	34.8	57.6

*PAD* pulmonary artery diameter, *ABS* aortic diameter at the base of the valve, *ASV* aortic diameter at the sinus of Valsalva, *AJT* aortic diameter at the sino-tubular junction, *MVD* mitral valve diameter, *LAD* left atrium diameter, *AOD* aortic diameter, *PEP* pre-ejection time, *ET* ejection time, *EPSS* septal-E point distance, *RVID* right ventricular internal diameter, *IVS* interventricular septal thickness, *LVID* left ventricular internal diameter, *LVFW* left ventricular free-wall thickness, *FS* fractional shortening, *d* diastole, *s* systole

**Table 2:** Results of multivariate linear regression of echocardiographic measurements in mixed-breed donkeys

Measurement	Parameter	*B*	Standard error	t	*p*-value
PAD (mm)	Intercept	17.026	2.405	7.079	< 0.001
	BW	0.070	0.011	6.250	**< 0.001**
	Age	-0.13	0.127	-0.102	0.919
	Sex	0.572	0.934	0.612	0.544
	Training	0.209	1.938	0.108	0.914
ABS (mm)	Intercept	20.938	1.584	13.216	< 0.001
	BW	0.063	0.007	8.502	**< 0.001**
	Age	0.101	0.084	1.199	0.237
	Sex	0.816	0.615	1.326	0.192
	Training	1.111	1.276	0.870	0.389
ASV (mm)	Intercept	25.310	1.694	14.938	< 0.001
	BW	0.070	0.008	8.869	**< 0.001**
	Age	0.171	0.090	1.905	0.064
	Sex	0.396	0.658	0.602	0.551
	Training	2.562	1.365	1.877	0.068
AJT (mm)	Intercept	21.146	1.875	11.279	< 0.001
	BW	0.055	0.009	6.266	**< 0.001**
	Age	0.205	0.099	2.069	0.051
	Sex	-0.107	0.728	-0.147	0.884
	Training	3.975	1.510	2.632	**0.012**
AOD (mm)	Intercept	25.356	2.033	12.475	< 0.001
	BW	0.070	0.009	7.496	**< 0.001**
	Age	0.077	0.106	0.728	0.471
	Sex	-0.079	0.809	-0.098	0.923
	Training	3.540	1.614	2.194	**0.034**
PEP (ms)	Intercept	110.490	14.822	7.455	< 0.001
	BW	-0.037	0.068	-0.551	0.585
	Age	-0.252	0.770	-0.327	0.746
	Sex	1.487	5.900	0.252	0.802
	Training	13.320	11.768	1.132	0.265
ET (ms)	Intercept	366.733	34.269	10.702	< 0.001
	BW	0.126	0.156	0.808	0.424
	Age	-0.889	1.780	-0.500	0.620
	Sex	21.397	13.640	1.569	0.125
	Training	-7.925	27.209	-0.291	0.772
EPSS (mm)	Intercept	2.802	0.911	3.074	0.004
	BW	-0.002	0.004	-0.494	0.624
	Age	0.007	0.048	0.149	0.882
	Sex	-0.046	0.354	-0.130	0.897
	Training	0.598	0.734	0.814	0.420
RVIDd (mm)	Intercept	20.574	4.018	5.121	< 0.001
	BW	-0.017	0.022	-0.759	0.456
	Age	0.138	0.179	0.771	0.449
	Sex	1.875	1.160	1.617	0.120
	Training	8.925	3.239	2.756	**0.012**
RVIDs (mm)	Intercept	9.832	4.710	2.088	0.049
	BW	0.013	0.026	0.492	0.628
	Age	-0.044	0.214	-0.205	0.839
	Sex	1.326	1.375	0.964	0.346
	Training	4.197	3.889	1.079	0.293
IVSd (mm)	Intercept	9.125	1.479	6.168	< 0.001
	BW	0.037	0.007	5.290	**< 0.001**
	Age	-0.012	0.078	-0.151	0.881
	Sex	1.579	0.575	2.748	**0.009**
	Training	-0.935	1.192	-0.784	0.438
IVSs (mm)	Intercept	16.779	2.309	7.268	< 0.001
	BW	0.056	0.011	5.219	**< 0.001**
	Age	0.179	0.122	1.460	0.152
	Sex	-0.277	0.897	-0.308	0.759
	Training	2.202	1.860	1.184	0.243
LVIDd (mm)	Intercept	35.825	3.578	10.012	< 0.001
	BW	0.134	0.017	8.002	**< 0.001**
	Age	-0.221	0.190	-1.165	0.251
	Sex	2.375	1.390	1.708	0.095
	Training	3.919	2.883	1.360	0.182
LVIDs (mm)	Intercept	17.320	3.403	5.089	< 0.001
	BW	0.068	0.016	4.272	**< 0.001**
	Age	-0.187	0.180	-1.039	0.305
	Sex	2.913	1.322	2.203	**0.033**
	Training	1.541	2.742	0.562	0.577
LVFWd (mm)	Intercept	9.831	1.391	7.068	< 0.001
	BW	0.020	0.007	3.056	**0.004**
	Age	0.112	0.074	1.523	0.136
	Sex	0.394	0.540	0.729	0.471
	Training	1.318	1.121	1.176	0.246
LVFWs (mm)	Intercept	19.525	2.411	8.099	< 0.001
	BW	0.011	0.011	1.004	0.322
	Age	0.134	0.128	1.046	0.302
	Sex	-0.377	0.937	-0.402	0.690
	Training	6.274	1.942	3.230	**0.002**
FS (%)	Intercept	49.711	3.700	13.435	< 0.001
	BW	0.003	0.017	0.153	0.879
	Age	0.105	0.196	0.534	0.597
	Sex	-2.236	1.437	-1.556	0.128
	Training	0.639	2.981	0.214	0.831
MVD (mm)	Intercept	40.312	4.041	9.977	< 0.001
	BW	0.072	0.019	3.823	**< 0.001**
	Age	0.259	0.214	1.208	0.234
	Sex	0.700	1.570	0.446	0.658
	Training	8.603	3.255	2.643	**0.012**
LAD (mm)	Intercept	45.336	4.156	10.909	< 0.001
	BW	0.093	0.019	4.797	**< 0.001**
	Age	0.108	0.220	0.490	0.627
	Sex	1.163	1.614	0.721	0.475
	Training	6.335	3.348	1.892	0.066

The echocardiographic measurements for which a statistically significant linear correlation was found are identified in bold

*BW* bodyweight, *PAD* pulmonary artery diameter, *ABS* aortic diameter at the base of the valve, *ASV* aortic diameter at the sinus of Valsalva, *AJT* aortic diameter at the sino-tubular junction, *AOD* aortic diameter, *PEP* pre-ejection time, *ET* ejection time, *EPSS* septal-E point distance, *RVID* right ventricular internal diameter, *IVS* interventricular septal thickness, *LVID* left ventricular internal diameter, *LVFW* left ventricular free-wall thickness, *FS* fractional shortening, *MVD* mitral valve diameter, *LAD* left atrium diameter, *d* diastole, s systole

**Table 3:** Intercept, slope, and coefficient of determination of the linear regression between echocardiographic measurements and body weight, and their 95% confidence intervals (minimum and maximum values)

Measurement	*R*	*R* ^2^	*P* value	Mean value		Minimum value		Maximum value	
				**a**	**b**	**a**	**b**	**a**	**b**
PAD (mm)	0.80	0.64	< 0.001	17.37	0.073	13.52	0.056	21.22	0.090
ABS (mm)	0.88	0.78	< 0.001	21.40	0.072	18.72	0.060	24.08	0.083
ASV (mm)	0.89	0.80	< 0.001	24.91	0.084	21.96	0.071	27.86	0.097
AJT (mm)	0.84	0.70	< 0.001	19.72	0.072	16.39	0.058	23.04	0.087
AOD (mm)	0.87	0.76	< 0.001	23.79	0.083	20.36	0.068	27.22	0.098
IVSd (mm)	0.73	0.53	< 0.001	10.91	0.039	8.34	0.027	13.48	0.050
IVSs (mm)	0.78	0.60	< 0.001	16.01	0.066	12.21	0.050	19.81	0.083
LVIDd (mm)	0.86	0.74	< 0.001	35.16	0.153	28.85	0.125	41.48	0.180
LVIDs (mm)	0.69	0.48	< 0.001	18.43	0.081	12.506	0.055	24.34	0.107
LVFWd (mm)	0.64	0.41	< 0.001	9.87	0.028	7.53	0.018	12.21	0.038
MVD (mm)	0.73	0.54	< 0.001	37.41	0.110	30.26	0.078	44.56	0.141
LAD (mm)	0.77	0.60	< 0.001	43.49	0.122	36.41	0.091	50.56	0.153

*R* coefficient of determination, *a* intercept, *b* slope, *PAD* pulmonary artery diameter, *ABS* aortic diameter at the base of the valve, *ASV* aortic diameter at the sinus of Valsalva, *AJT* aortic diameter at the sino-tubular junction, *AOD* aortic diameter, *IVS* interventricular septal thickness, *LVID* left ventricular internal diameter, *LVFW* left ventricular free-wall thickness, *MVD* mitral valve diameter, *LAD* left atrium diameter, *d* diastole, *s* systole

## Discussion

The present study reported a strong linear correlation between echocardiographic measurements and bodyweight in mixed-breed donkeys. Weak or no correlation were found between cardiac measurement, sex and age. While the results of the multivariate regression analysis suggested that training may influence some echocardiographic measurements.

To the best of the authors’ knowledge, this is the first study to investigate the influence of training on echocardiographic measurements in donkeys. In horses has been demonstrated that training affects cardiac dimensions (Buhl et al. 2005). Training is also an important variable to consider in donkeys, as these animals are employed in working and sports activities. However, only 12 donkeys included in this study underwent regular training for sport activity. Therefore, further studies with a larger number of racing donkeys would be necessary to better evaluate the effect of training on cardiac dimensions in this species.

Multivariate linear regression revealed a lack of correlation between echocardiographic measurements and age, in agreement with previous studies conducted on donkeys, suggesting that this variable should not be considered when assessing echocardiographic measurements in this species (Amory et al. 2004; Hassan and Torad 2015; Roberts and Dukes-McEwan 2016b; Farag and Ibrahim 2020). However, some studies conducted on horses reported a correlation between some echocardiographic measurements and age, particularly in growing animals (Lombard et al. 1984). In the present study, young donkeys were included (age range: 8 months – 24 years); however, newborn donkeys were not evaluated. Therefore, further future studies could be conducted to assess the possible influence of age on echocardiographic measurements in newborn animals.

Sex showed a weak but statistically significant correlation only with IVSd and LVIDs. However, due to the weakness of this relationship, it is unlikely to be of clinical importance. The absence of a clinically significant correlation with sex was in accordance with previous studies conducted on equids (Al-Haidar et al. 2013; Roberts and Dukes-McEwan 2016b; Farag and Ibrahim 2020).

In the literature, it has been reported that bodyweight influences cardiac dimensions in small animals and horses and should be considered when defining reference ranges for echocardiographic measurements (Al-Haidar et al. 2013, 2017; Vatne et al. 2021). In the present study, body weight strongly affected most of the echocardiographic measurements. This is in contrast with previous studies carried out on donkeys, in which the association between BW and cardiac measurements was weak or not present at all (Hassan and Torad 2015; Roberts and Duke-McEwan 2016b; Farag and Ibrahim 2020). This finding could be due to the lower sample size (Hassan and Torad 2015: 30; Roberts and Duke-McEwan 2016b: 36; Farag and Ibrahim 2020: 44) and the narrow body weight ranges (Hassan and Torad 2015: 150–220 kg; Roberts and Duke-McEwan 2016b: 130–262 kg; Farag and Ibrahim 2020: 150–350 kg) reported in these studies. An association between BW and cardiac dimensions has already been reported by Amory and colleagues (2004), however many of their measurements were acquired from different echocardiographic views compared to those used in this study, and therefore not comparable.

**Fig. 3:**
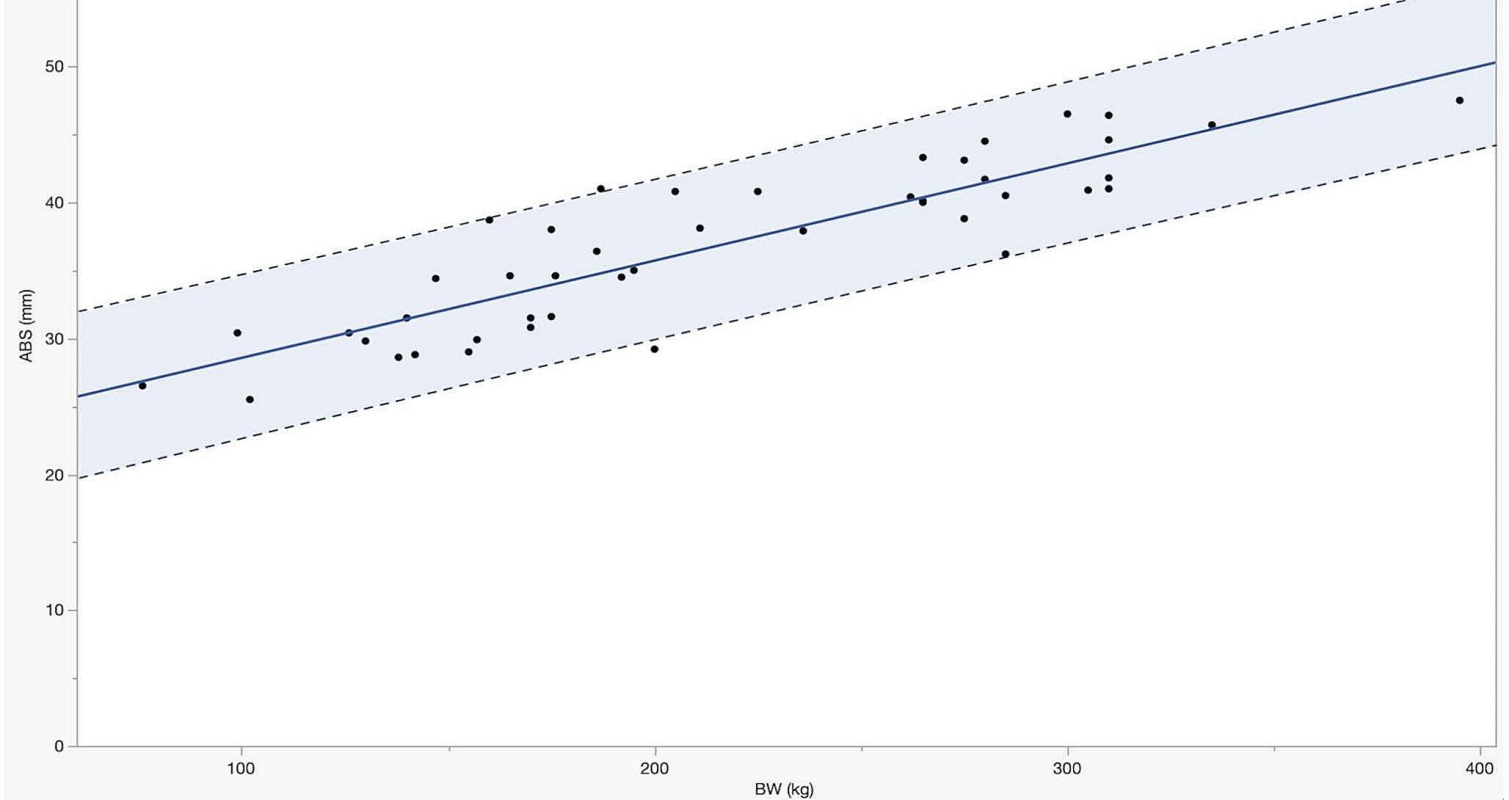
Linear regression of the end-diastolic aortic diameter at the base of the valve (ABS) versus body weight (BW), showing the regression line and the 95% prediction interval for this echocardiographic measurement. The continuous line shows the equation of the mean values (ABS = 0.072*BW + 21.40) and the dotted lines show the maximum (ABS = 0.083*BW + 24.08) and the minimum (ABS = 0.060*BW + 18.72) prediction intervals values of the ABS

Since a highly statistically significant correlation between most of the echocardiographic measurements and BW was found, a linear regression model was used to describe this relationship. The results obtained in the present study provided a simple linear equation that can be used to predict echocardiographic measurements as a function of BW in donkeys with a wide range of body weight. A representation of the linear regression of the end-diastolic aortic diameter at the base of the valve (ABS) versus body weight is shown in Fig. 3.

Three jennies were excluded from the study because in the first months of pregnancy when the echocardiographic examination was performed. In humans and small animals, it has been reported that pregnancy affects cardiac dimensions (Abbott 2010; Iloeje et al. 2023), whereas cardiac remodeling was not detected in a study carried out on horses (Chompoosan et al. 2023). Therefore, further studies could be conducted on pregnant jennies to assess possible changes in echocardiographic measurements.

This study had some possible limitations. First, the animals included in the present study were characterized by a wide range of age and bodyweight. However, this variability is highly representative of the heterogeneous donkey population in Italy. Second, the bodyweight of the donkeys was not measured but was only estimated using the Donkey Sanctuary’s diagram, that associates the heart girth and the height measurements (Svendsen 2008b). However, this could also be considered an advantage since donkeys are usually evaluated in the field, where a weighing scale is barely available. Third, since all the donkeys had a BCS of 3 out of 5, the influence of BCS on echocardiographic measurements was not evaluated. Therefore, further studies should be carried out to determine the possible effect of BW on echocardiographic measurements in underweight and overweight animals. Last, since the echocardiography was performed by a single examiner in each donkey, interobserver echocardiographic repeatability was not assessed in the present study.

In conclusion, this is the first study reporting a strong linear correlation between echocardiographic measurements and bodyweight, and suggesting that training may influence cardiac measurements in mixed-breed donkeys. This last variable should be further investigated to assess its meaning for cardiac examination in donkeys.

## Data Availability

The data analyzed during the current study are available from the corresponding author upon reasonable request.
